# Prediction of the development of acute kidney injury following cardiac surgery by machine learning

**DOI:** 10.1186/s13054-020-03179-9

**Published:** 2020-07-31

**Authors:** Po-Yu Tseng, Yi-Ting Chen, Chuen-Heng Wang, Kuan-Ming Chiu, Yu-Sen Peng, Shih-Ping Hsu, Kang-Lung Chen, Chih-Yu Yang, Oscar Kuang-Sheng Lee

**Affiliations:** 1 Institute of Clinical Medicine, School of Medicine, National Yang-Ming University, No. 155, Section 2, Li-Nong Street, Beitou District, Taipei, 11221 Taiwan; 2 Stem Cell Research Center, National Yang-Ming University, Taipei, Taiwan; 3 Division of Nephrology, Department of Internal Medicine, Taipei City Hospital, Heping Fuyou Branch, Taipei, Taiwan; 4 Muen Biomedical and Optoelectronics Technologies Inc., New Taipei City, Taiwan; 5 Division of Cardiovascular Surgery, Cardiovascular Center, Far Eastern Memorial Hospital, New Taipei City, Taiwan; 6 Department of Electrical Engineering, Yuan Ze University, Taoyuan City, Taiwan; 7 Division of Nephrology, Department of Internal Medicine, Far Eastern Memorial Hospital, New Taipei City, Taiwan; 8 College of Electrical and Communication Engineering, Yuan Ze University, Taoyuan City, Taiwan; 9 Department of Applied Cosmetology, Lee-Ming Institute of Technology, New Taipei City, Taiwan; 10 Division of Cardiovascular Surgery, En Chu Kong Hospital, New Taipei City, Taiwan; 11 Division of Nephrology, Department of Medicine, Taipei Veterans General Hospital, Taipei, Taiwan; 12 Center for Intelligent Drug Systems and Smart Bio-devices (IDS2B), Hsinchu, Taiwan; 13 China Medical University Hospital, Taichung, Taiwan

**Keywords:** Cardiac surgery, Acute kidney injury, Machine learning, Prediction

## Abstract

**Background:**

Cardiac surgery–associated acute kidney injury (CSA-AKI) is a major complication that results in increased morbidity and mortality after cardiac surgery. Most established prediction models are limited to the analysis of nonlinear relationships and fail to fully consider intraoperative variables, which represent the acute response to surgery. Therefore, this study utilized an artificial intelligence–based machine learning approach thorough perioperative data-driven learning to predict CSA-AKI.

**Methods:**

A total of 671 patients undergoing cardiac surgery from August 2016 to August 2018 were enrolled. AKI following cardiac surgery was defined according to criteria from Kidney Disease: Improving Global Outcomes (KDIGO). The variables used for analysis included demographic characteristics, clinical condition, preoperative biochemistry data, preoperative medication, and intraoperative variables such as time-series hemodynamic changes. The machine learning methods used included logistic regression, support vector machine (SVM), random forest (RF), extreme gradient boosting (XGboost), and ensemble (RF + XGboost). The performance of these models was evaluated using the area under the receiver operating characteristic curve (AUC). We also utilized SHapley Additive exPlanation (SHAP) values to explain the prediction model.

**Results:**

Development of CSA-AKI was noted in 163 patients (24.3%) during the first postoperative week. Regarding the efficacy of the single model that most accurately predicted the outcome, RF exhibited the greatest AUC (0.839, 95% confidence interval [CI] 0.772–0.898), whereas the AUC (0.843, 95% CI 0.778–0.899) of ensemble model (RF + XGboost) was even greater than that of the RF model alone. The top 3 most influential features in the RF importance matrix plot were intraoperative urine output, units of packed red blood cells (pRBCs) transfused during surgery, and preoperative hemoglobin level. The SHAP summary plot was used to illustrate the positive or negative effects of the top 20 features attributed to the RF. We also used the SHAP dependence plot to explain how a single feature affects the output of the RF prediction model.

**Conclusions:**

In this study, machine learning methods were successfully established to predict CSA-AKI, which determines risks following cardiac surgery, enabling the optimization of postoperative treatment strategies to minimize the postoperative complications following cardiac surgeries.

## Introduction

Cardiac surgery–associated acute kidney injury (CSA-AKI) is a complication following cardiac surgery and is associated with increased morbidity and mortality as well as prolonged hospital stay and higher medical costs [1, 2]. One meta-analysis of global incidence and outcomes of CSA-AKI during the period 2004–2014 indicated that the incidence was approximately 22% for all stages of AKI. The pooled short- and long-term mortality rates were 10.7% and 30%, respectively, and increased with the severity of AKI [1]. Even a slight increase in serum creatinine after cardiac surgery is related to a significant increase in 30-day mortality [3].

The pathophysiology of CSA-AKI is multifactorial and remains incompletely understood. Hypoperfusion, ischemia-reperfusion injury, neurohormonal activation, inflammation, nephrotoxin exposure, and cardiopulmonary bypass (CPB)–related nonpulsatile perfusion are known to be contributing factors [4, 5]. All of the aforementioned events may occur perioperatively [6]. To appropriately manage CSA-AKI, a precise prediction model for identifying high-risk patients is required to optimize the postoperative treatment strategy. Several previously published prediction models have shown reasonable ability to discriminate patients with the risk of severe AKI or AKI requiring dialysis [7–14]. However, the definitions of AKI in these models have been inconsistent, and only a handful of models have used intraoperative variables, which may critically affect the prediction of AKI. No unified definition of AKI has been reported in the literature until the development of Risk, Injury, Failure, Loss, End-Stage Kidney Disease (RIFLE) and Acute Kidney Injury Network (AKIN) criteria [15]. The Kidney Disease: Improving Global Outcomes (KDIGO) criteria for AKI staging is modified by AKIN and demonstrates more sensitive AKI detection [16]. The first model for the prediction of all AKI stages, including less severe stage 1 cases, was developed using consensus KDIGO criteria in a prospective study [17]. All risk models were developed using the logistic regression method, which requires the statistical assumption of a linear relationship between the variables and outcome. Moreover, logistic regression requires independent variables and selects small subsets of input variables based on their statistical significance for multiple regression models. But some variables that have causal effects on the output variable may not be statistically significant [18]. We might reduce the available information and miss unexpected relationships that could be utilized to improve predictive power if we excluded the variables only due to statistical assumptions.

To analyze numerous variables with nonlinearity and complex relationships that may be associated with CSA-AKI development, an alternative and effective approach is required for the development of precise prediction models. Machine learning has been applied in areas of medicine such as outcome prediction, diagnosis, medical image interpretation, and treatment [19, 20]. Machine learning techniques require no assumptions regarding input variables and their relationships with the output. The advantage of completely data-driven learning without reliance on rules-based programming is that machine learning constitutes a reasonable approach. Therefore, this study applied machine learning methods to develop a model for the accurate prediction of CSA-AKI. Preoperative variables and intraoperative time-series physiological data were used to optimize the prediction model.

## Methods

### Study population

We retrospectively reviewed the medical records of 671 patients who underwent coronary artery bypass (CABG), valve replacement surgery, and a combination of both treatments at Far Eastern Memorial Hospital (FEMH), New Taipei City, from August 2016 to August 2018. Institutional Review Board approval from FEMH (106159-E) was obtained prior to the commencement of this study, and informed consent was waived because the research involved no more than minimal risk to patients. The waiver does not adversely affect the rights and welfare of the participants.

### Data collection and preprocessing of data

**Fig. 1:**
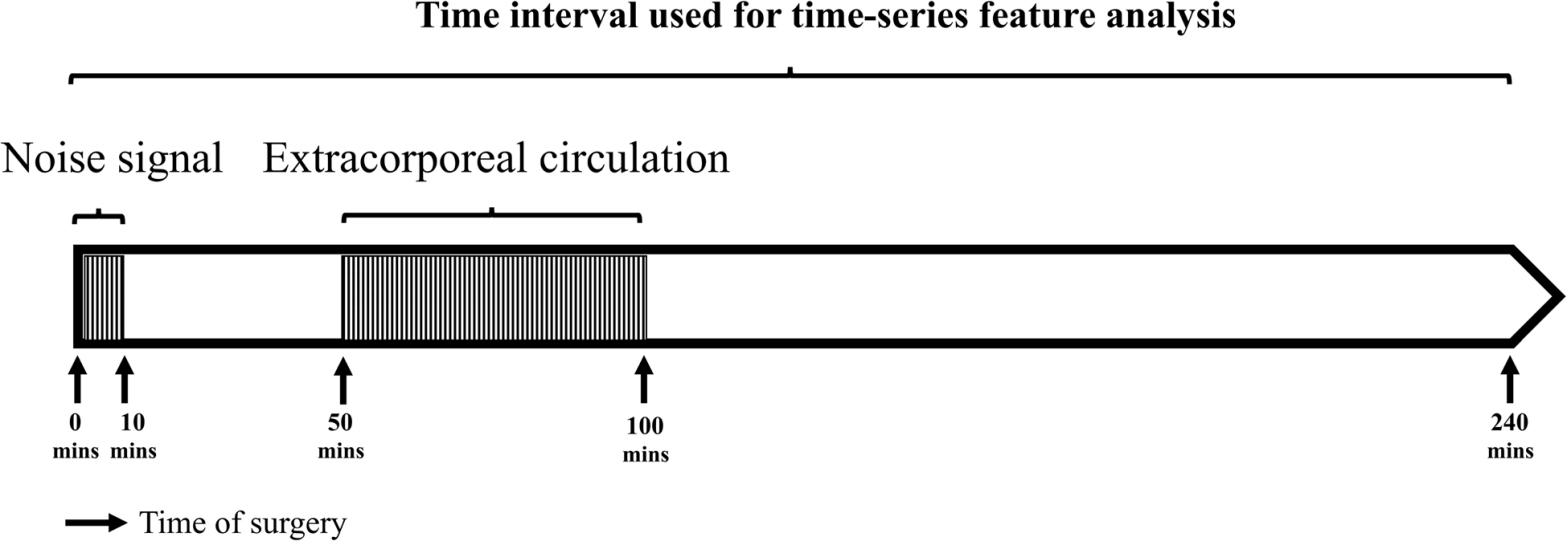
Time period obtained during operation for ARV calculation. We obtained data for the 240 min after operation except the initial 10 min (due to noise signals) and the period between 50 and 100 min after operation due to extracorporeal circulation

We collected data on demographic characteristics, clinical condition, preoperative biochemistry data, preoperative medication, and intraoperative time-series hemodynamic features (systolic blood pressure [SBP], diastolic blood pressure [DBP], mean arterial blood pressure [MAP], and heart rate [HR]) from electronic medical records and records on intraoperative variables at FEMH. All data except for the time-series features were collected through manual review of the medical records. Time-series data were obtained from electronic records saved at the database. Patients with an estimated glomerular filtration rate (eGFR) < 60 mL/min/1.73 m^2^ for more than 3 months were defined as having chronic kidney disease (CKD). Furthermore, eGFR was calculated for all patients using the Chronic Kidney Disease Epidemiology Collaboration creatinine (CKD-EPI) equation [21]. For the time-series features (SBP, DBP, HR), the 240-min period after the beginning of the operation was used for analysis. The mean arterial pressure (MAP) was calculated using the equation: MAP = DBP + 0.01exp (4.14–40.74/HR) (SBP-DBP) [22]. We also used the average real variability (ARV) index to represent short-term, reading-to-reading, within-subject variability in blood pressure [23]; this provided a more accurate estimator of variance compared with other measures of dispersion, including standard deviation (SD), coefficient of variation (CV), and weighted SD [24]. Before ARV calculation, we excluded data for 10 min after the operation began because of excessive noise signals and data from 50 to 100 min after the operation because many patients underwent extracorporeal circulation during that time interval (Fig. 1). The other time-series data comprised 180 readings. We calculated the ARVs of SBP, DBP, and HR using the following formula:

$$ \mathrm{ARV}=\frac{1}{\sum Wk}{\sum}_{K=2}^n{W}_k\times \mid {BP}_k-{BP}_{k-1}\mid . $$

where *n* is the number of BP readings, and *W*_*k*_ is the time interval between *BP*_*k*_ and *BP*_*k−1*_*.* A total of 179 real variabilities existed for the time-series features in each patient. We used principal component analysis (PCA) to reduce the dimensionality from 179 to 10 for the real variability (RV) data calculated using SBP, DBP, and HR. We directly used PCA to reduce the dimensionality of the absolute value of MAP instead of RV. Moreover, we used the maximal RVs of time-series features as predictive variables.

Among all the variables, the overall rate of missing data was 0.16%. The missing data were inputted as the average values or modes for the variables.

### Definition of cardiac surgery–associated acute kidney injury

Development of postoperative AKI was defined according to KDIGO criteria during the first 7 days after operation [16]. Postoperative AKI was defined as either at an increase of at least 50% within 7 days or 0.3 mg/dL elevation within 48 h compared with the reference serum creatinine level. The serum creatinine level measured before surgery was used as the reference value.

### Machine learning

The data were randomly divided, with 70% used for training and 30% for validation. To overcome the imbalance of data in the training set, we copied the positive cases 5 times to prevent overfitting. All analyses were developed in Python (version 3.5). We attempted the following supervised machine learning methods to develop the predictive models, which are the most popular and up-to-date machine learning methods used for the problem of classification: logistic regression, simple decision tree, random forest (RF), support vector machine (SVM), eXtreme Gradient Boosting (XGboost), and ensemble (RF + XGboost). The logistic regression model accurately predicts the probability of the binary dependent variable using maximum likelihood estimation to determine the regression coefficient. Tree-based learning algorithms include the simple decision tree, RF, and XGboost. A decision tree method is a tree-like model of decisions that can predict the best choice mathematically. We used an optimized version of the Classification and Regression Trees algorithm to develop the simple decision tree [25]. We used the Gini index as a metric to identify the split point. The Gini index is the probability of randomly classify incorrectly in the dataset. The weakness of the simple decision tree is instability and a risk of overfitting, and thus RF and XGboost are created to improve the prediction. RF is an ensemble classifier that combines multiple decision trees through majority voting [26, 27]. XGboost is an optimized distributed gradient boosting library that provides superior prediction through the conversion of a set of weak learners to strong learners. The algorithm is powerful by some innovations, such as the approximate greedy search, parallel learning, and hyperparameters [28]. We also attempted SVM, an algorithm for identifying a high-dimensional boundary that distinctly classifies data points.

To evaluate the prediction and accuracy of various machine learning models, we calculated and compared areas under the receiver operating characteristic curve (AUC). The correct interpretation of a prediction model for machine learning is a challenge. We used SHapley Additive exPlanation (SHAP) values to provide consistent and locally accurate attribution values for each feature within each prediction model [29]. This is a unified approach for explaining the outcome of any machine learning model. SHAP values evaluate the importance of the output resulting from the inclusion of feature A for all combinations of features other than A.

## Results

We reviewed the medical records of 671 patients undergoing cardiac surgery from August 2016 to August 2018. The demographics and perioperative variables are listed in Table 1. We divided the patients randomly and allocated 70% of them to the training set and the remaining 30% to the test set. Among the patients, 250 (37.3%) received CABG, 347 (51.7%) received valve replacement surgery, and 74 (11%) underwent combined CABG and valve surgery. For the time-series features (SBP, DBP, MAP, HR), we calculated the ARVs of the time-series features, and the data are listed in Table 1. For all variables, the differences between the training set and the test set were nonsignificant. The clinical characteristics and perioperative variables for patients who developed CSA-AKI or did not are listed in Additional file 1.

**Table 1:** Patient characteristics and perioperative variables

**Variables**	**All**	**Training set**	**Test set**	***p*value**
**Patient population,*n***	671	469	202	
**Demographic data**				
Age (years)	63 (53–70)	62 (53–69)	63 (53–71)	0.323
Male, *n* (%)	454 (67.7)	323 (68.9)	131 (64.9)	0.307
BMI (kg/m^2^)	24.8 (22.5–27.8)	24.8 (22.5–27.6)	24.8 (22.7–27.4)	0.956
Allergy, *n* (%)	598 (89.1)	42 (9.0)	31 (15.3)	0.015
ABO, *n* (%)				
Type A	196 (29.2)	135 (28.8)	61 (30.2)	0.715
Type B	144 (21.5)	110 (23.5)	34 (16.8)	0.055
Type AB	42 (6.3)	30 (6.4)	12 (5.9)	0.823
Type O	289 (43.1)	194 (41.4)	95 (47.0)	0.174
Smoking, *n* (%)	244 (36.4)	171 (36.5)	73 (36.1)	0.937
**Surgery type**				
CABG only, *n* (%)	250 (37.3)	166 (35.4)	84 (41.6)	0.128
Valve surgery only, *n* (%)	347 (51.7)	246 (52.5)	101 (50.0)	0.560
Combined CABG + valve surgery, *n* (%)	74 (11.0)	57 (12.2)	17 (8.4)	0.156
Minimally invasive surgery, *n* (%)	336 (50.1)	237 (50.5)	99 (49.0)	0.717
**Preoperative condition**				
ER stay > 1 day, *n* (%)	59 (8.8)	42 (9.0)	17 (8.4)	0.821
Previous cardiac surgery, *n* (%)	96 (14.3)	59 (12.6)	37 (18.3)	0.052
Preoperative ventilator support, *n* (%)	45 (6.7)	33 (7.0)	12 (5.9)	0.603
Preoperative IABP, *n* (%)	19 (2.8)	10 (2.1)	9 (4.5)	0.096
Cardiogenic shock, *n* (%)	24 (3.6)	14 (3.0)	10 (5.0)	0.209
Arrhythmia, *n* (%)	138 (20.6)	93 (19.8)	45 (22.3)	0.472
**Medical history**				
Family history of CAD, *n* (%)	80 (11.9)	59 (12.6)	21 (10.4)	0.423
Diabetes mellitus, *n* (%)	215 (32)	152 (32.4)	63 (31.2)	0.756
Dyslipidemia, *n* (%)	258 (38.5)	177 (37.7)	81 (40.1)	0.564
HTN, *n* (%)	453 (67.5)	313 (66.7)	140 (69.3)	0.515
TIA, *n* (%)	23 (3.4)	13 (2.8)	10 (5.0)	0.155
Cerebrovascular accident, *n* (%)	39 (5.8)	27 (5.8)	12 (5.9)	0.926
Infective endocarditis, *n* (%)	34 (5.1)	21 (4.5)	13 (6.4)	0.289
COPD, *n* (%)	73 (10.9)	49 (10.4)	24 (11.9)	0.584
PAOD, *n* (%)	17 (2.5)	13 (2.8)	4 (2.0)	0.549
Chronic kidney disease, *n* (%)	207 (30.8)	136 (29.0)	71 (35.1)	0.114
Congestive heart failure, *n* (%)	131 (19.5)	93 (19.8)	38 (18.8)	0.760
Myocardial infarction, *n* (%)	104 (15.5)	70 (14.9)	34 (16.8)	0.531
**Baseline laboratory findings**				
Serum creatinine (mg/dL)	0.91 (0.71–1.16)	0.90 (0.70–1.14)	0.95 (0.73–1.28)	0.128
eGFR (CKD-EPI: mL/min/1.73 m^2^)	78.0 (53.2–99.7)	80.6 (55.6–100.9)	73.0 (47.4–98.4)	0.057
Hgb (g/dL)	13.2 (11.4–14.6)	13.3 (11.5–14.6)	13.1 (11.4–14.3)	0.403
Preoperative LVEF (%)	62.0 (47.0–69.0)	62.0 (49.0–70.0)	61.0 (44.0–68.3)	0.323
LVEDD (mm)	51.6 (46.0–56.0)	51.6 (46.0–56.0)	51.6 (45.0–56.0)	0.864
Number of diseased coronary vessels, *n* (%)				
One vessel disease	68 (10.1)	48 (10.2)	20 (9.9)	0.896
Two vessel disease	59 (8.8)	40 (8.5)	19 (9.4)	0.713
Three vessel disease	255 (38.0)	175 (37.3)	80 (39.6)	0.575
CCS Angina Score, *n* (%)				
Score 0	125 (18.6)	88 (18.8)	37 (18.3)	0.892
Score 1	134 (20.0)	97 (20.7)	37 (18.3)	0.482
Score 2	356 (53.1)	243 (51.8)	113 (55.9)	0.326
Score 3	50 (7.5)	37 (7.9)	13 (6.4)	0.511
Score 4	6 (0.9)	4 (0.9)	2 (1.0)	0.862
NYHA functional class, *n* (%)				
Class I	113 (16.8)	84 (17.9)	29 (14.4)	0.259
Class II	365 (54.4)	251 (53.5)	114 (56.5)	0.486
Class III	161 (24.0)	109 (23.2)	52 (25.7)	0.486
Class IV	32 (4.8)	25 (5.3)	7 (3.5)	0.298
ASA Physical Status Classification, *n* (%)				
Class I	10 (1.5)	7 (1.5)	3 (1.5)	0.994
Class II	119 (17.7)	84 (17.9)	35 (17.3)	0.856
Class III	485 (72.3)	337 (71.9)	148 (73.3)	0.708
Class IV	51 (7.6)	38 (8.1)	13 (6.4)	0.455
**Preoperative medications**				
Beta blockers, *n* (%)	297 (44.3)	212 (45.2)	85 (42.1)	0.455
ACEi, *n* (%)	68 (10.1)	51 (10.9)	17 (8.4)	0.333
ARB, *n* (%)	138 (20.6)	93 (19.8)	45 (22.3)	0.472
Intravenous nitroglycerin, *n* (%)	20 (3.0)	14 (3.0)	6 (3.0)	0.992
Anticoagulants, *n* (%)	162 (24.1)	105 (22.4)	57 (28.2)	0.106
Steroids, *n* (%)	26 (3.9)	19 (4.1)	7 (3.5)	0.718
Aspirin, *n* (%)	181 (27.0)	127 (27.1)	54 (26.7)	0.926
Lipid-lowering agents, *n* (%)	268 (39.9)	185 (39.4)	83 (41.1)	0.690
Dobutamin, *n* (%)	7 (1.0)	4 (0.9)	3 (1.5)	0.460
OHA, *n* (%)	162 (24.1)	115 (24.5)	47 (23.3)	0.728
Insulin, *n* (%)	39 (5.8)	22 (4.7)	17 (8.4)	0.059
**Intraoperative variables**				
Operation time (mins)	225.0 (190.0–270.0)	225.0 (195.0–270.0)	220.0 (180.0–261.3)	0.101
Elective, *n* (%)	625 (93.1)	439 (93.6)	186 (92.1)	0.577
Robotic technology assisted, *n* (%)	7 (1.0)	4 (0.9)	3 (1.5)	0.460
Cross clamp time (mins)	44.0 (0.0–70.0)	46.0 (0.0–70.5)	40.5 (0.0–67.3)	0.179
Perfusion time (mins)	95.0 (0.0–132.0)	97.0 (0.0–136.0)	93.0 (0.0–124.3.0)	0.096
Lowest core temperature (°C)	33.0 (32.0–37.0)	32.0 (32.0–37.0)	34.0 (32.0–37.0)	0.704
Cardioversion, *n* (%)	192 (28.6)	131 (27.9)	61 (30.2)	0.551
Intraoperative IV fluid infusion (mL/kg/h)	11.2 (8.4–15.2)	11.0 (8.4–15.2)	11.8 (9.0–15.2)	0.302
Intraoperative urine output (mL/kg/h)	2.2 (1.2–3.8)	2.2 (1.3–3.7)	2.2 (1.2–4.0)	0.765
Intraoperative estimated blood loss (mL/kg/h)	2.5 (1.7–3.6)	2.5 (1.7–3.5)	2.6 (1.7–3.7)	0.547
Cardiopulmonary bypass utilization, *n* (%)	471 (70.2)	336 (71.6)	135 (66.8)	0.211
pRBC transfusion during surgery (units)	3.0 (0.0–6.0)	3.0 (0.0–6.0)	4.0 (0.0–6.25)	0.302
FFP transfusion during surgery (units)	0.0 (0.0–3.0)	0.0 (0.0–3.0)	0.0 (0.0–3.0)	0.979
PLT transfusion during surgery (units)	0.0 (0.0–0.0)	0.0 (0.0–0.0)	0.0 (0.0–6.0)	0.151
**Time-series features (ARV)**				
ARV of HR (beats/min)	3.7 (2.7–5.2)	3.7 (2.7–5.2)	3.5 (2.5–5.3)	0.979
ARV of SBP (mmHg)	7.3 (5.9–8.7)	7.3 (5.9–8.7)	7.3 (5.8–8.9)	0.736
ARV of DBP (mmHg)	4.7 (3.7–6.1)	4.8 (3.7–6.1)	4.6 (3.6–5.9)	0.819
ARV of MAP (mmHg)	5.5 (4.3–6.7)	5.5 (4.3–6.7)	5.3 (4.3–6.6)	0.889
**AKI according to KDIGO criteria**	163 (24.3)	114 (24.3)	49 (24.3)	0.989
Stage I, *n* (%)	119 (17.7)	87 (18.6)	32 (15.8)	0.399
Stage II, *n* (%)	20 (3.0)	14 (3.0)	6 (3.0)	0.992
Stage III, *n* (%)	24 (3.6)	13 (2.8)	11 (5.4)	0.087

Data are presented as median (interquartile range) or number (%). *Abbreviations*: *BMI* body mass index, *CABG* coronary artery bypass grafting, *ER* emergency room, *CAD* coronary artery disease, *HTN* hypertension, *TIA* transient ischemic stroke, *COPD* chronic obstructive pulmonary disease, *PAOD* peripheral artery occlusive disease, *eGFR* estimated glomerular filtration rate, *CKD-EPI* Chronic Kidney Disease Epidemiology Collaboration, *LVEF* left ventricular ejection fraction, *LVEDD* left ventricular end-diastolic diameter, *CCS* Canadian Cardiovascular Society, *NYHA* New York Heart Association, *ASA* American Society of Anesthesiologists, *ACEi* angiotensin-converting-enzyme inhibitor, *ARB* angiotensin II receptor blocker, *OHA* oral hypoglycemic agents, *IV* intravenous, *pRBC* packed red blood cell, *FFP* fresh frozen plasma, *PLT* platelet, *ARV* average real variability, *HR* heart rate, *SBP* systolic blood pressure, *DBP* diastolic blood pressure, *MAP* mean arterial pressure, *AKI* acute kidney injury, *KDIGO* kidney disease improving global outcome

**Fig. 2:**
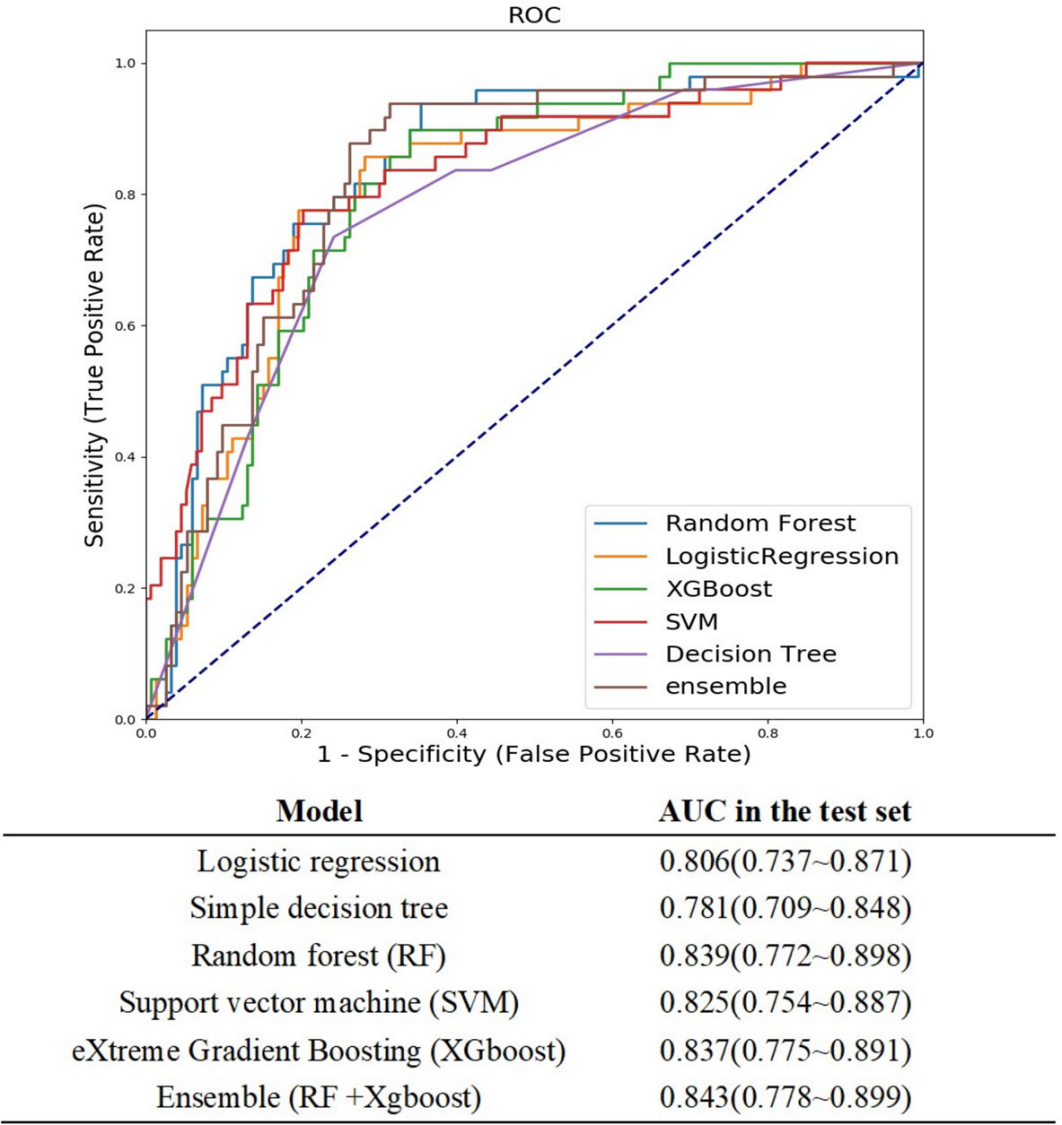
Comparison of AUCs among machine learning models. RF yielded the greatest AUC for single-model prediction. The AUC for RF + XGboost was even greater than for the RF model alone

We utilized the following machine learning methods with all the variables as input variables, including logistic regression, simple decision tree, RF, SVM, XGboost, and RF + XGboost to predict postoperative AKI, and the AUCs are presented in Fig. 2. Regarding the efficacy of the single model for outcome prediction, RF exhibited the largest AUC (0.839, 95% confidence interval [CI] 0.772–0.898). The AUC (0.843, 95% CI 0.778–0.899) for the ensemble model was larger than that for the RF model alone. The simple decision tree exhibited the smallest AUC (0.78, 95% CI 0.71–0.85).

**Fig. 3:**
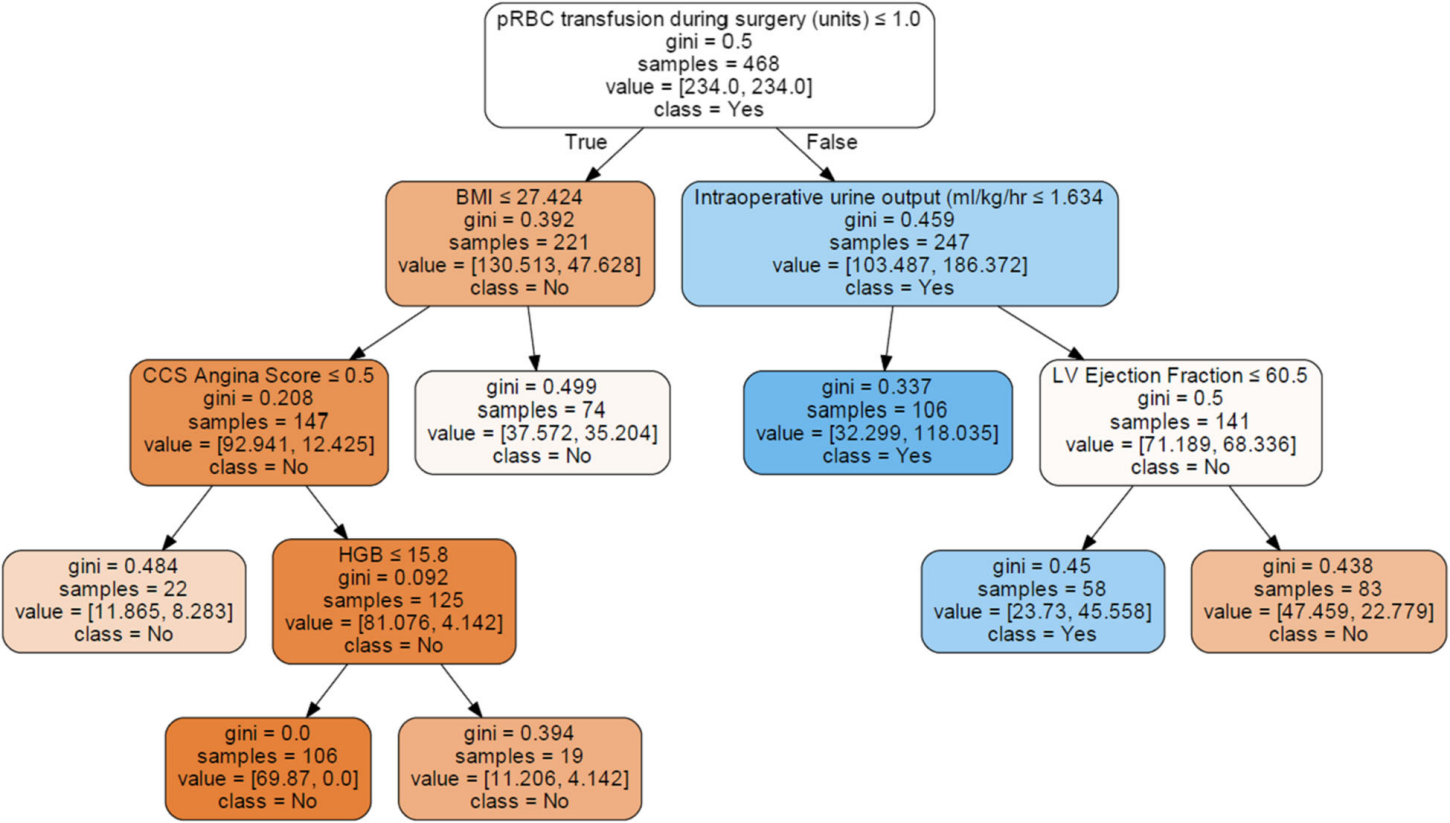
Simple decision tree model illustrating the classification of patients with (class = yes) and without (class = no) acute kidney injury. Each box has the following components: selected variables for classification, Gini index, number of samples classified to the box according to the previous variable, the average number of patients for each classification with 5-cross validation, and the majority of classes at the split node. Blue and orange represent the yes class and the no class, respectively, and the color densities increase when the Gini indexes decrease. Abbreviations: pRBC, packed red blood cell; BMI, body mass index; CCS, Canadian Cardiovascular Society; LV, left ventricular; HGB, hemoglobin

Figure 3 presents a simple decision tree model for classifying patients into with or without AKI. The Gini index in the terminal leaf exceeded 0.45 in 3 of 7 leaf nodes, which implied that the classification was inaccurate.

**Fig. 4:**
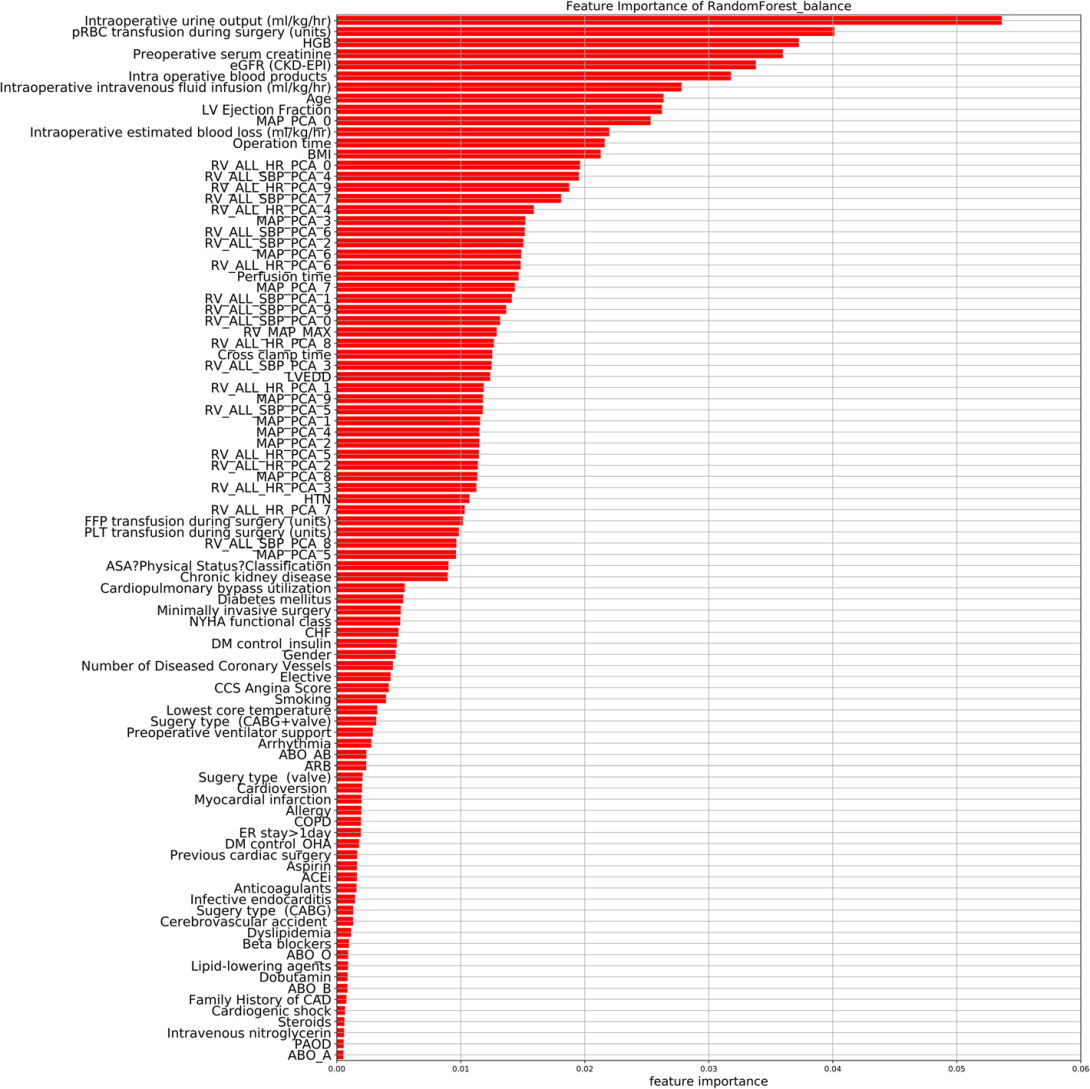
Importance matrix plot of the RF model. This importance matrix plot depicts the importance of each covariate in the development of the final predictive model. Abbreviations: HGB, hemoglobin; eGFR, estimated glomerular filtration rate; CKD-EPI, Chronic Kidney Disease Epidemiology Collaboration; LV, left ventricular; MAP, mean arterial pressure; PCA, principal component analysis; BMI, body mass index; RV, real variability; HR, heart rate; SBP, systolic blood pressure; LVEDD, left ventricular end-diastolic diameter; HTN, hypertension; FFP, fresh frozen plasma; PLT, platelet; ASA, American Society of Anesthesiologists; CHF, congestive heart failure; DM, diabetes mellitus; CCS, Canadian Cardiovascular Society; CABG, coronary artery bypass grafting; ARB, angiotensin II receptor blocker, COPD, chronic obstructive pulmonary disease; OHA, oral hypoglycemic agent; ER, emergency room; ACEi, angiotensin-converting-enzyme inhibitor; CAD, coronary artery disease; PAOD, peripheral artery occlusive disease

The importance matrix plot for the RF method is shown in Fig. 4 and reveals that the top 5 most important variables contributing to the model were intraoperative urine output, pRBC transfusion during surgery, preoperative hemoglobin (HGB), preoperative serum creatinine, and preoperative eGFR. Of the top 20 most important features, 14 were intraoperative variables and 8 were time-series variables.

**Fig. 5:**
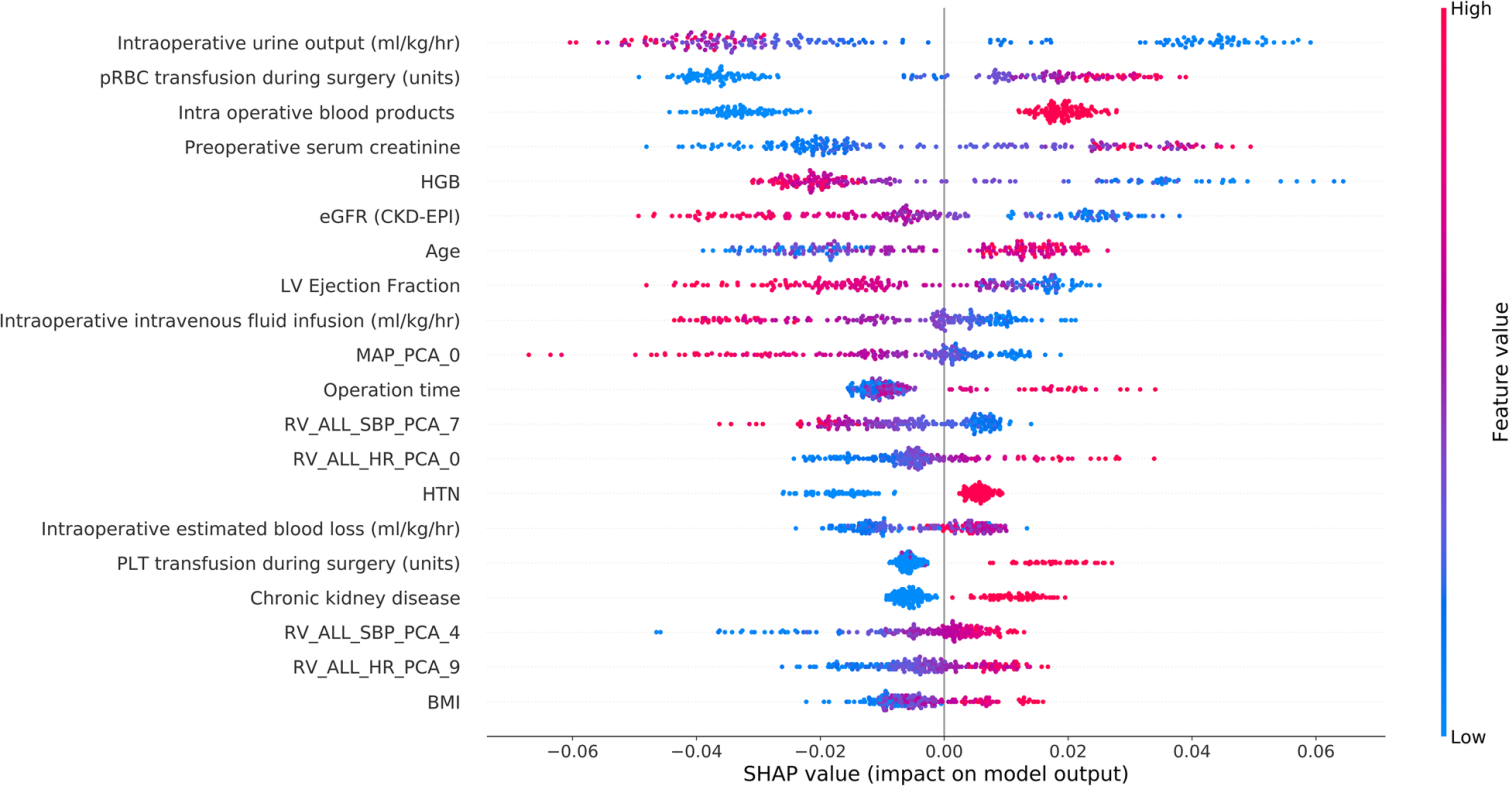
SHAP summary plot of the top 20 features of the RF model. The higher the SHAP value of a feature, the higher the probability of postoperative acute kidney injury development. A dot is created for each feature attribution value for the model of each patient, and thus one patient is allocated one dot on the line for each feature. Dots are colored according to the values of features for the respective patient and accumulate vertically to depict density. Red represents higher feature values, and blue represents lower feature values. Abbreviations: pRBC, packed red blood cell; HGB, hemoglobin; eGFR, estimated glomerular filtration rate; CKD-EPI, Chronic Kidney Disease Epidemiology Collaboration; LV, left ventricular; MAP, mean arterial pressure; PCA, principal component analysis; SBP, systolic blood pressure; HR, heart rate; RV, real variability; HTN, hypertension; PLT, platelet; BMI, body mass index

**Fig. 6:**
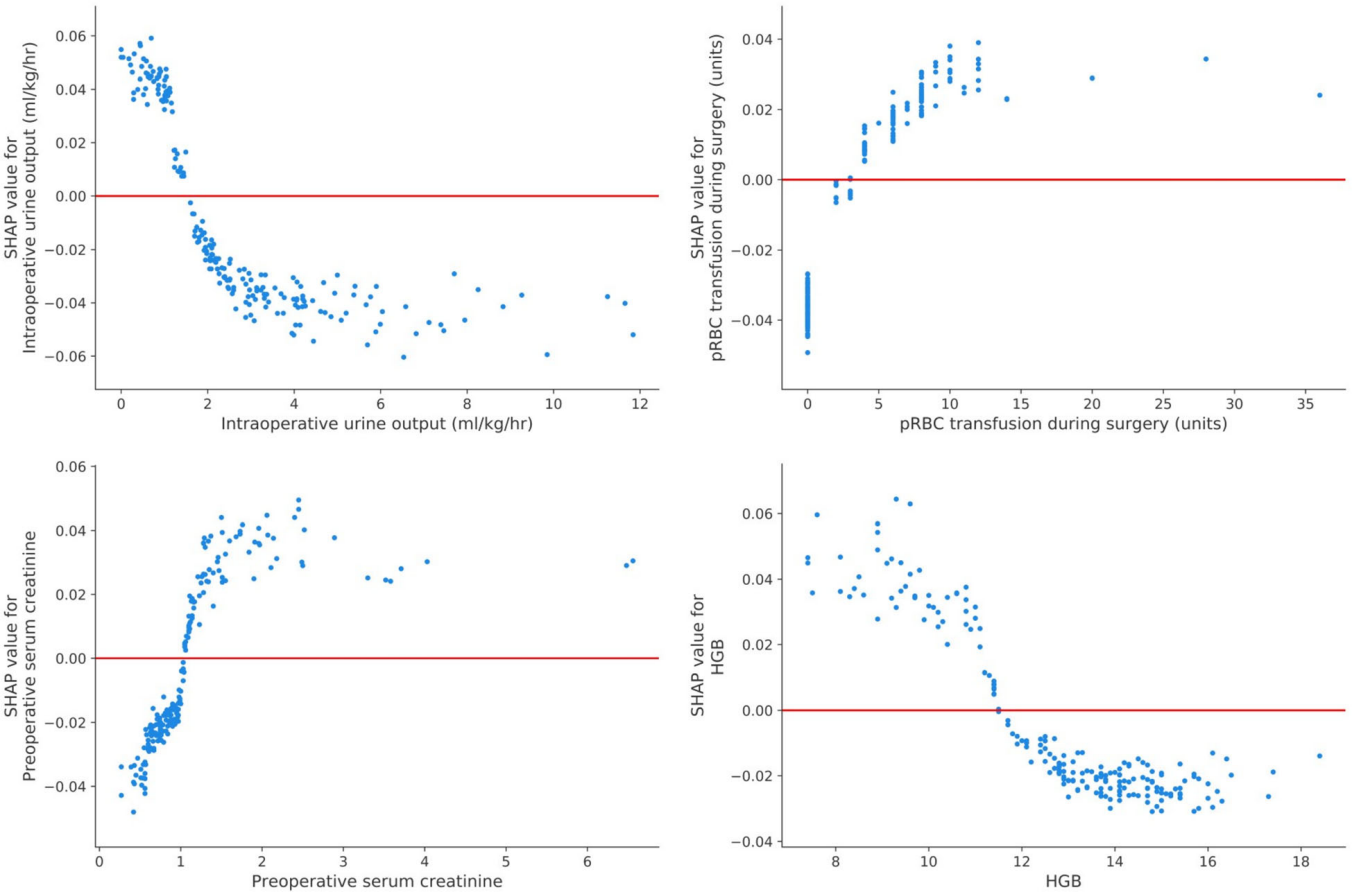
SHAP dependence plot of the RF model. The SHAP dependence plot shows how a single feature affects the output of the RF prediction model. SHAP values for specific features exceed zero, representing an increased risk of acute kidney injury development. Abbreviations: pRBC, packed red blood cell; HGB, hemoglobin; eGFR, estimated glomerular filtration rate; CKD-EPI, Chronic Kidney Disease Epidemiology Collaboration

To identify the features that influenced the prediction model the most, we depicted the SHAP summary plot of RF (Fig. 5) and the top 20 features of the prediction model. This plot depicts how high and low features’ values were in relation to SHAP values in the training dataset. According to the prediction model, the higher the SHAP value of a feature, the more likely AKI becomes. The SHAP dependence plot (Fig. 6) can also be used to understand how a single feature affects the output of the RF prediction model. The *y*-axis values indicated the SHAP values of features, and the values of features for the *x*-axis were in the SHAP dependence plot. We could visualize how the feature’s attributed importance changed as its values varied in the plot. SHAP values for specific features exceeding zero represent an increased risk of AKI development.

## Discussion

In this retrospective cohort study, we developed and validated machine learning algorithms using 94 preoperative and intraoperative features to predict CSA-AKI. The RF model exhibited the best performance for single-model prediction, whereas the RF + XGboost model exhibited the greatest AUC among the models we tested. The RF and XGboost models are bootstrapping method applications, which can improve the predictive power when available datasets are small. Over half of the top 20 features on the importance matrix plot and the SHAP summary plot of RF were intraoperative features, which implies that the major effects of intraoperative condition on early kidney function decline following cardiac surgery. This study demonstrated the value of intraoperative data, which reflected acute physiological responses during surgery relevant to CSA-AKI prediction; the previously used prediction models emphasized preoperative conditions.

Established risk scores for AKI prediction following cardiac surgery were reviewed by Huen et al. and classified into AKI requiring dialysis and severe AKI according to a broad definition. Four clinical risk scores for AKI prediction requiring dialysis, including the Continuous Improvement in Cardiac Surgery Study score [8], the Cleveland Clinic Score [9], the Mehta score [10], and the Simplified Renal Index score [11], were developed using only preoperative variables. Another 3 clinical scores—the Multicenter Study of Perioperative Ischemia Score [12], the Acute Kidney Injury After Cardiac Surgery Score [13], and the Northern New England Cardiovascular Disease Study Group Score [14]—were developed to predict severe AKI. Two of these enrolled some intraoperative variables for the generation of postoperative AKI risk scores. Most of the studies were analyzed with the multivariable logistic regression method, with the performance regarding AUC ranging from 0.76 to 0.84. None used the currently accepted definitions for AKI. One prospective study of more than 30,000 patients in the UK used KDIGO criteria for CSA-AKI prediction [17]. This model demonstrated improved discrimination compared with the Cleveland Clinic Score and similar discrimination to the Mehta score.

The first study in the literature to use a machine learning approach for CSA-AKI at all stages of prediction reported that the optimal AUC was achieved with XGboost (0.78, 95% CI 0.75–0.80) [30]. The study demonstrated that the performances of machine learning models were significantly superior to those of traditional logistic regression models for the prediction of AKI following cardiac surgery. In addition, the study revealed that the AUCs of the previously used risk score models were often only 0.55 in their datasets, potentially due to the small numbers of predictors used and the lack of intraoperative variables. Our SHAP summary plot for RF exhibited some similar predictors known to be associated with CSA-AKI according to traditional risk score models. However, our plot revealed additional novel predictors, some of which were consistent with the importance matrix plot of gradient boosting in the previous machine learning study. Moreover, 5 of the top 20 features that contributed to the model obtained through the use of a SHAP summary plot were time-series variables, which were not analyzed by the aforementioned study but may have improved the AUC in our research. One single-center cohort study proposed a machine learning algorithm to reclassify approximately 40% of patients undergoing any surgery, who were considered to be at low risk of AKI by a preoperative model but were reclassified as high risk after the inclusion of intraoperative features [31]. This also proved that intraoperative features play a major role in AKI risk stratification. In their study, intraoperative time-series features were processed into the minimum, maximum, mean, short-term, and long-term variability [32], which may lead to the loss of useful information compared with our method of PCA for time-series features to preserve as much of the variability in the original data as possible.

The reasons we used ARV and RV to represent time-series features variability are as follows: There are three indexes that were commonly used to evaluate 24-h blood pressure (BP) variability (BPV) in the literature. The first one is the standard deviation (SD) of 24-h ambulatory BP monitoring recordings, which accounts only for the dispersion of values around the mean, not considering the ordering of BP readings [33]. Because the SD is correlated with mean BP, it can be inadequate in a multivariate prognostic model if more than two different measurements were used. The second one is the coefficient of variation (CV), which is the solution to overcome the above problem of SD [34]. Another major weakness of the 24-h SD is that its value is significantly affected by the nocturnal BP decrease. The third index is “weighted” 24-h SD (wSD), which is the average of diurnal and nocturnal SDs by weighting for their respective durations. It can minimize the effect of nocturnal dipping without losing the information on BPV [35]. However, no standardized methods exist for the accurate estimation of BPV. According to the published systemic review and meta-analysis, ARV is a more accurate estimator of 24-h BPV [24]. Despite using ARV in our study, we also used PCA of RV to reserve the most information of the time-series features variability.

The advantage of our study is the use of SHAP values to uncover the black box of machine learning. Although several risk factors have been identified by previously used risk score models, such as preoperative HGB, preoperative renal function, age, operation time, left ventricular ejection fraction, body mass index, and hypertension [7–14], the recognition of intraoperative urine output, IV fluid infusion, blood product transfusion, and dynamic changes of hemodynamic features are important risk factors that have been neglected by traditional risk score models. Notably, some well-known risk factors were not ranked among the top 20 features in our study, such as diabetes mellitus, CPB time, and surgery type. The pathophysiology of CSA-AKI may explain why intraoperative features are so crucial to AKI prediction. Although the definite mechanism is not completely elucidated, renal hypoperfusion is known to result from low-flow, low-pressure, nonpulsatile perfusion with hemodilution; moreover, rapid temperature reduction because of CPB usage, bleeding complications, and inflammatory response play vital roles in CSA-AKI development [4]. Hemodynamic change, blood product transfusion, IV fluid supplement, and intraoperative urine output all reflect the acute response for renal hypoperfusion and the management required. The risk of AKI following cardiac surgery was determined by the preoperative health condition–related susceptibility to acute stress and large dynamic physiological responses intraoperatively, reflecting the ongoing response to surgery. Therefore, software may be developed that can identify high-risk patients who are prone to AKI for the optimization of treatment strategies after cardiac surgery. Moreover, extremely few values are missing from the dataset because most of the data were recorded by hand. Therefore, missing values would not have negatively affected the results.

This study was subject to some limitations. First, our analysis used only single-center data and included relatively few patients. The performance of the machine learning algorithm might differ for larger datasets with differently distributed patient characteristics and different institutions. As such, external validation is required to prevent overfitting. Second, the algorithm learned from the input features, and some hidden relationships may have been lost because of unknown or neglected features that were not enrolled by physicians. Third, most of the input features were achieved manually. We are working on developing a real-time automated electronic health record algorithm that can aggregate perioperative information of patients from various data sources. With these techniques, a machine learning–based predictive model may have the potential for use in clinical practice. Fourth, we used PCA to reduce the dimensionality of time-series features instead of analyzing the original data because of the small numbers of participants in this cohort study, which may have led to the loss of chronological and implied information. Deep learning methods might be used for numerous time-series features if more patients are enrolled. Fifth, we did not use the previous risk scores for performance comparison because of the unavailability of all the variables required in the previously used risk score models. Lastly, predictive ability was impaired by the relatively small numbers of positive events resulting from data imbalance. Future prospective studies are required to evaluate the application of machine learning–based predictive models to clinical practice for the reduction of AKI risks.

## Conclusions

In conclusion, we successfully applied the machine learning method to predict AKI after cardiac surgery, which can be used to determine risks after surgery. We demonstrated that the intraoperative time-series and other features are crucial for AKI prediction. Further software development is ongoing for the real-time adjustment of AKI risks following cardiac surgery, which in turn will optimize treatment to improve prognosis.

## Supplementary information

**Additional file 1.** Characteristics and perioperative variables of patients developed acute kidney injury or not after cardiac surgery.

## Data Availability

Non-applicable
