# Supplementary material for: Prediction of the development of acute kidney injury following cardiac surgery by machine learning
Source: Crit Care. 2020 Jul 31;24:478. doi: 10.1186/s13054-020-03179-9 (PMC7395374; doi:10.1186/s13054-020-03179-9)
Supplement: Supplementary file 1 — Additional file 1. Characteristics and perioperative variables of patients developed acute kidney injury or not after cardiac surgery. [file 13054_2020_3179_MOESM1_ESM.pdf]

Table 1. Characteristics and perioperative variables of patients developed acute kidney injury or not after cardiac surgery

Variables	All	AKI	No AKI	p-Value
**Patient population, n**	671	163	508	
**Demographic data**				
Age (years)	63 (53-70)	66 (59-73)	61 (51-69)	0.000*
Male, n (%)	454 (67.7)	121 (74.2)	333 (65.6)	0.039*
BMI (kg/m^2)	24.8 (22.5-27.8)	25.2 (23.0-28.2)	24.7 (22.4-27.3)	0.187
Allergy, n (%)	598 (89.1)	18 (11.0)	55 (10.8)	0.939
ABO, n (%)				
Type A	196 (29.2)	48 (29.4)	148 (29.1)	0.939
Type B	144 (21.5)	31 (19.0)	113 (22.2)	0.383
Type AB	42 (6.3)	14 (8.6)	28 (5.5)	0.158
Type O	289 (43.1)	70 (42.9)	219 (43.1)	0.970
Smoking, n (%)	244 (36.4)	75 (46.0)	169 (33.2)	0.003*
**Surgery type**				
CABG only, n (%)	250 (37.3)	51 (31.3)	199 (39.2)	0.070
Valve surgery only, n (%)	347 (51.7)	76 (46.6)	271 (53.3)	0.135
Combined CABG + Valve surgery, n (%)	74 (11.0)	36 (22.1)	38 (7.4)	0.000*
Minimally invasive surgery, n (%)	336 (50.1)	59 (36.2)	277 (54.5)	0.000*
**Preoperative condition**				
ER stay >1 day, n (%)	59 (8.8)	27 (16.6)	32 (6.3)	0.000*
Previous cardiac surgery, n (%)	96 (14.3)	33 (20.2)	63 (12.4)	0.013*
Preoperative ventilator support, n (%)	45 (6.7)	22 (13.5)	23 (4.5)	0.000*
Preoperative IABP, n (%)	19 (2.8)	8 (4.9)	11 (2.2)	0.066
Cardiogenic shock, n (%)	24 (3.6)	12 (7.4)	12 (2.4)	0.003*
Arrhythmia, n (%)	138 (20.6)	43 (26.4)	95 (18.7)	0.035*
**Medical history**				
Family history of CAD, n (%)	80 (11.9)	18 (11.0)	62 (12.2)	0.690
Diabetes mellitus, n (%)	215 (32)	75 (46.0)	140 (27.6)	0.000*
Dyslipidemia, n (%)	258 (38.5)	69 (42.3)	189 (37.2)	0.242
HTN, n (%)	453 (67.5)	136 (83.4)	317 (62.4)	0.000*
TIA, n (%)	23 (3.4)	8 (4.9)	15 (3.0)	0.233
Cerebrovascular accident, n (%)	39 (5.8)	15 (9.2)	24 (4.7)	0.033*
Infective endocarditis, n (%)	34 (5.1)	11 (6.7)	23 (4.5)	0.261
COPD, n (%)	73 (10.9)	27 (16.6)	46 (9.1)	0.007*
PAOD, n (%)	17 (2.5)	9 (5.5)	8 (1.6)	0.005*

Chronic kidney disease, n (%)	207 (30.8)	84 (51.5)	123 (24.2)	0.000*
Congestive heart failure, n (%)	131 (19.5)	53 (32.5)	78 (15.4)	0.000*
Myocardial infarction, n (%)	104 (15.5)	36 (22.1)	68 (13.4)	0.008*
**Baseline laboratory findings**				
Serum creatinine (mg/dL)	0.91 (0.71-1.16)	1.1 (0.8-1.5)	0.9 (0.7-1.1)	0.000*
eGFR (CKD-EPI: mL/min/1.73m^2)	78.0 (53.2-99.7)	58.0 (38.2-84.5)	84.0 (61.6-102.4)	0.000*
Hgb (g/dL)	13.2 (11.4-14.6)	12.3 (10.0-14.0)	13.5 (12.0-14.7)	0.000*
Preoperative LVEF (%)	62.0 (47.0-69.0)	33.0 (37.0-66.0)	63.0 (53.0-70.0)	0.000*
LVEDD (mm)	51.6 (46.0-56.0)	51.6 (48.0-58.0)	51.6 (46.0-55.0)	0.010*
Number of Diseased Coronary Vessels, n (%)				
One vessel disease	68 (10.1)	17 (10.4)	51 (10.0)	0.886
Two vessel disease	59 (8.8)	18 (11.0)	41 (8.1)	0.244
Three vessel disease	255 (38.0)	73 (44.8)	182 (35.8)	0.040*
CCS Angina Score, n (%)				
Score 0	125 (18.6)	46 (28.2)	79 (15.6)	0.000*
Score 1	134 (20.0)	21 (12.9)	113 (22.2)	0.009*
Score 2	356 (53.1)	79 (48.5)	277 (54.5)	0.177
Score 3	50 (7.5)	15 (9.2)	35 (6.9)	0.328
Score 4	6 (0.9)	2 (1.2)	4 (0.8)	0.604
NYHA functional class, n (%)				
Class I	113 (16.8)	30 (18.4)	83 (16.3)	0.540
Class II	365 (54.4)	69 (42.3)	296 (58.3)	0.000*
Class III	161 (24.0)	49 (30.1)	112 (22.0)	0.037*
Class IV	32 (4.8)	15 (9.2)	17 (3.3)	0.002*
ASA Physical Status Classification, n (%)				
Class I	10 (1.5)	2 (1.2)	8 (1.6)	0.750
Class II	119 (17.7)	20 (12.3)	99 (19.5)	0.036*
Class III	485 (72.3)	115 (70.6)	370 (72.8)	0.571
Class IV	51 (7.6)	22 (13.5)	29 (5.7)	0.002*
**Preoperative medications**				
Beta blockers, n (%)	297 (44.3)	64 (39.3)	233 (45.9)	0.140
ACEi, n (%)	68 (10.1)	19 (11.7)	49 (9.6)	0.459
ARB, n (%)	138 (20.6)	25 (15.3)	113 (22.2)	0.058
Intravenous nitroglycerin, n (%)	20 (3.0)	5 (3.1)	15 (3.0)	0.940
Anticoagulants, n (%)	162 (24.1)	48 (29.4)	114 (22.4)	0.069
Steroids, n (%)	26 (3.9)	8 (4.9)	18 (3.5)	0.432
Aspirin, n (%)	181 (27.0)	58 (35.6)	123 (24.2)	0.004*
Lipid-lowering agents, n (%)	268 (39.9)	68 (41,7)	200 (39.4)	0.594

Dobutamin, n (%)	7 (1.0)	1 (0.6)	6 (0.1)	0.535
OHA, n (%)	162 (24.1)	54 (33.1)	108 (21.3)	0.020*
Insulin, n (%)	39 (5.8)	20 (12.3)	19 (3.7)	0.000*
**Intraoperative variables**				
Operation time (mins)	225.0 (190.0-270.0)	240.0 (195.0-300.0))	220.0 (180.0-260.0)	0.002*
Elective, n (%)	625 (93.1)	141 (86.5)	484 (95.3)	0.000*
Robotic technology assisted, n (%)	7 (1.0)	1 (0.6)	6 (0.1)	0.535
Cross clamp time (mins)	44.0 (0.0-70.0)	47.0 (0.0-75.0)	43.0 (0.0-69.0)	0.041*
Perfusion time (mins)	95.0 (0.0-132.0)	108.0 (77.0-149.0)	92.0 (0.0-131.0)	0.000*
Lowest Core Temperature (°C)	33.0 (32.0-37.0)	32.0 (32.0-37.0)	34.0 (32.0-37.0)	0.041*
Cardioversion, n (%)	192 (28.6)	63 (38.7)	129 (25.4)	0.001*
Intraoperative IV fluid infusion (mL/kg/hr)	11.2 (8.4-15.2)	9.8 (7.7-12.8)	11.8 (8.7-15.6)	0.000*
Intraoperative urine output (mL/kg/hr)	2.2 (1.2-3.8)	1.4 (0.8-2.4)	2.2 (1.3-3.7)	0.000*
Intraoperative estimated blood loss (mL/kg/hr)	2.5 (1.7-3.6)	3.0 (2.2-4.1)	2.4 (1.6-3.4)	0.000*
Cardiopulmonary bypass utilization, n (%)	471 (70.2)	139 (85.3)	332 (65.4)	0.000*
pRBC transfusion during surgery (units)	3.0 (0.0-6.0)	6.0 (4.0-10.0)	0.0 (0.0-5.0)	0.000*
FFP transfusion during surgery (units)	0.0 (0.0-3.0)	0.0 (0.0-4.0)	0.0 (0.0-0.0)	0.000*
PLT transfusion during surgery (units)	0.0 (0.0-0.0)	0.0 (0.0-12.0)	0.0 (0.0-0.0)	0.000*
**Time-series features (ARV)**				
ARV of HR (beats/min)	3.7 (2.7-5.2)	4.4 (2.8-5.7)	3.6 (2.7-5.1)	0.002*
ARV of SBP (mmHg)	7.3 (5.9-8.7)	7.4 (5.7-8.8)	7.3 (5.9-8.7)	0.648
ARV of DBP (mmHg)	4.7 (3.7-6.1)	4.8 (3.6-6.1)	4.7 (3.7-6.1)	0.947
ARV of MAP (mmHg)	5.5 (4.3-6.7)	5.3 (4.2-6.7)	5.5 (4.3-6.7)	0.638

Data are presented as median (interquartile range) or number (%). * means p-value < 0.05 when comparing patients with and without acute kidney injury following cardiac surgery. Abbreviations: AKI, acute kidney injury; BMI, body mass index; CABG, coronary artery bypass grafting; ER, emergency room; CAD, coronary artery disease; HTN, hypertension; TIA, transient ischemic stroke; COPD, chronic obstructive pulmonary disease; PAOD, peripheral artery occlusive disease; eGFR, estimated glomerular filtration rate; CKD-EPI, Chronic Kidney Disease Epidemiology Collaboration, LVEF = left ventricular ejection fraction; LVEDD, left ventricular end-diastolic diameter; CCS, Canadian Cardiovascular Society; NYHA, New York Heart Association; ASA, American Society of Anesthesiologists; ACEi, angiotensin-converting-enzyme inhibitor; ARB, angiotensin II receptor blocker; OHA, oral hypoglycemic agents; IV, intravenous; pRBC, packed red blood cell; FFP, fresh frozen plasma; PLT, platelet; ARV, average real variability; HR, heart rate; SBP, systolic blood pressure, DBP, diastolic blood pressure; MAP, mean arterial pressure.
